# Clinical, Neuroimaging, and Genetic Markers Associated with Cognitive and Functional Outcomes After Traumatic Brain Injury

**DOI:** 10.3390/jcm14082796

**Published:** 2025-04-18

**Authors:** Khrystyna Duve, Svitlana Shkrobot, Pavlo Petakh, Valentyn Oksenych, Oleksandr Kamyshnyi

**Affiliations:** 1 Department of Neurology, I. Horbachevsky Ternopil National Medical University, 46001 Ternopil, Ukraine; shkrobot@tdmu.edu.ua; 2 Department of Biochemistry and Pharmacology, Uzhhorod National University, 88000 Uzhhorod, Ukraine; pavlo.petakh@uzhnu.edu.ua; 3 Broegelmann Research Laboratory, Department of Clinical Science, University of Bergen, 5007 Bergen, Norway; 4 Department of Microbiology, Virology, and Immunology, I. Horbachevsky Ternopil National Medical University, 46001 Ternopil, Ukraine; kamyshnyi_om@tdmu.edu.ua

**Keywords:** chronic traumatic encephalopathy, traumatic brain injury, cognitive impairment, logistic regression

## Abstract

**Background**: Traumatic brain injury (TBI) is a major cause of long-term disability worldwide, often leading to progressive cognitive and functional impairments. This study aimed to investigate the underlying factors contributing to long-term deterioration in TBI patients. **Methods**: We conducted a comprehensive evaluation of 145 patients aged 18–66 years with a documented history of TBI and ongoing cognitive and behavioral deficits. Assessments included neuroimaging, laboratory tests, genetic analysis, and standardized tools such as the Montreal Cognitive Assessment (MoCA) and the Barthel Index. **Results**: Structural brain abnormalities, including ventricular enlargement and gliosis, were observed in a substantial portion of the cohort. Persistent neuroinflammatory markers were also identified. Genetic analysis revealed a significant association between cognitive decline and polymorphisms in the *ACE* and *PON1* genes. Patients carrying these variants were more likely to exhibit reduced cognitive performance and greater functional limitations. **Conclusion**: These findings suggest that genetic predisposition, chronic neuroinflammation, and structural brain damage collectively contribute to long-term outcomes following TBI. This highlights the potential of genetic and imaging biomarkers in identifying high-risk individuals and supports the need for personalized approaches to diagnosis, monitoring, and treatment in chronic TBI management.

## 1. Introduction

Encephalopathy is a term used to describe diverse types of brain dysfunction with varying causes and clinical presentations. It can arise as a secondary complication of other conditions or as a primary disorder. Traumatic brain injury (TBI), one of the leading causes of encephalopathy, accounts for 30–40% of all injuries globally and remains a major contributor to mortality and disability, particularly among socially and economically active individuals. Annually, 50–60 million people worldwide experience TBI, with projections suggesting it will remain one of the top three causes of trauma-related deaths and disabilities by 2030 [1,2].

In Ukraine, the impact of TBI has been exacerbated by armed conflict. Between 2014 and 2019, over 200,000 cases of TBI were reported, with numbers surging further due to the full-scale invasion in 2022. This critical increase has affected both military and civilian populations, although precise statistics remain unavailable. Diagnosing encephalopathy following TBI remains challenging worldwide due to the lack of clear diagnostic criteria that allow identification without postmortem pathological confirmation, as well as inconsistencies in terminology [3,4].

The condition caused by repeated head injuries was first described by H. Martland in 1928 as “punch-drunk syndrome”, emphasizing clinical manifestations such as cognitive and motor impairments [5,6]. In 1957, M. Critchley introduced the term “chronic progressive traumatic encephalopathy” (CTE), highlighting its neurodegenerative nature. A.C. McKee and her team significantly advanced the understanding of CTE in 2013 by defining it as a neuropathological diagnosis characterized by the accumulation of hyperphosphorylated tau protein in specific brain regions [7,8].

In 2014, P.H. Montenigro and colleagues proposed the term “traumatic encephalopathy syndrome” (TES) to describe clinical manifestations associated with repeated brain injuries [9]. However, the validity and specificity of TES as a clinical entity have been questioned. G. Iverson and J. Schaffert emphasized significant overlap between TES symptoms and other conditions, such as depression, which complicates its differentiation from other neuropsychiatric disorders [10,11]. These critiques highlight the need for further refinement of TES diagnostic criteria and additional research into its clinical relevance.

In 2015, a consensus conference organized by the National Institute of Neurological Disorders and Stroke (NINDS) defined neuropathological criteria for CTE, identifying hyperphosphorylated tau as its pathognomonic feature [12,13]. In 2021, Katz et al. proposed detailed guidelines for diagnosing TES in living patients, incorporating cognitive, behavioral, and motor symptoms alongside a history of repeated injuries and neuroimaging findings [13]. While these criteria represent progress, their application in routine clinical practice remains challenging and requires further validation.

Given the historical ambiguity and modern limitations of classifications, this study uses the term “patients with encephalopathy following traumatic brain injury”. This approach avoids terminological controversies and focuses on the clinical and radiological aspects of the condition.

The development and progression of encephalopathy in patients following TBI involve complex and interconnected mechanisms. A key pathological feature is the accumulation of hyperphosphorylated tau protein (p-tau) in neurons and glial cells, particularly in perivascular areas and cortical sulci. This contributes to neuronal toxicity, synaptic dysfunction, and brain circuit disruptions [14,15]. Chronic inflammation and prolonged activation of microglia further exacerbate neurodegeneration. Impairments in the reparative capacity of microglia and other neural pathways significantly affect the brain’s ability to recover, leading to cumulative neuronal loss and functional decline [16]. Additionally, disruption of the glymphatic system, which plays a critical role in clearing metabolic waste, accelerates the buildup of β-amyloid and other toxic proteins, worsening brain damage [17].

Cognitive decline in these patients is influenced by the severity of the injury, disturbances of consciousness, comorbidities, and age. Cognitive reserve serves a protective function, and its depletion is linked to faster disease progression. Clinical manifestations vary widely and encompass cognitive, behavioral, and motor symptoms. Cognitive impairments often include memory loss, executive dysfunction, and reduced attention [18,19,20,21,22].

Behavioral and emotional symptoms, such as aggression, impulsivity, depression, and anxiety, are common and can precede motor dysfunction [23,24].

Motor impairments are typical in advanced stages and include parkinsonism (tremor, mask-like facies, rigidity, and gait instability), dysarthria, dysphagia, coordination deficits, and statolocomotor ataxia. Early stages are often marked by chronic headaches, while motor neuron disease (chronic traumatic encephalomyelopathy) may develop later, presenting as muscle weakness, atrophy, spasticity, and fasciculations. These symptoms highlight the progressive and multifaceted nature of the condition [25,26].

Despite significant advancements in research, the mechanisms underlying encephalopathy in TBI patients remain incompletely understood. Key contributors include neuroinflammation, excitotoxicity, tau pathology, disruption of the glymphatic system responsible for waste clearance, and deficits in the brain’s reparative capacity, which collectively drive neurodegeneration and clinical decline [27,28].

The heterogeneity of clinical symptoms in encephalopathies following traumatic brain injury underscores the importance of further research in this area, particularly given that these patients are often young, socially, and professionally active. The lack of specific markers for the antemortem diagnosis of chronic post-traumatic encephalopathy underscores the importance of our study, which focuses on identifying gene polymorphisms significantly associated with cognitive impairments and functional impairment in this condition.

Consequently, the objective of this study was to examine clinical–neurological, neuroimaging, laboratory, and genetic factors potentially contributing to the progression of cognitive dysfunction and functional impairment in patients with encephalopathy following traumatic brain injury.

## 2. Materials and Methods

### 2.1. Study Design and Criteria

We conducted a prospective single-center cohort study at the Ternopil Regional Clinical Psychoneurological Hospital from 2021 to 2022. The study included patients aged 18 to 66 years with a history of traumatic brain injury (TBI) and clinical signs of encephalopathy, characterized by progressive cognitive, behavioral, and/or motor impairments associated with prior TBI. Patients included in the study demonstrated clinical, neurological, and neuroimaging evidence of encephalopathy progression. The inclusion process involved a retrospective analysis of each patient’s medical records combined with follow-up evaluations conducted after the initial traumatic brain injury to confirm the progression of symptoms.

The inclusion criteria required patients to be aged between 18 and 66 years and to have a documented history of traumatic brain injury (TBI), whether open or closed. The severity and type of TBI were determined by neurosurgeons during the acute period of TBI. Eligible participants presented with clinical and neurological evidence of encephalopathy, including neurological symptoms, cognitive impairment, behavioral changes, and neuroimaging abnormalities not attributable to any other underlying pathology. All patients demonstrated progressive neurological deterioration over the past one to two years.

The exclusion criteria include the presence of oncological conditions or decompensated somatic diseases, the use of medications affecting cognitive or memory functions (such as hypnotics, benzodiazepines, or antipsychotics) within the four weeks prior to inclusion, suspected Alzheimer’s disease or other neurodegenerative conditions, and evidence of alcohol abuse or psychoactive substance use.

### 2.2. Demographic and Clinical Variables

We analyzed key demographic variables, including sex, age, disease duration, employment status (working or not), physical or mental activity, and level of education, to assess their potential influence on the progression and clinical manifestations of encephalopathy following traumatic brain injury.

We also conducted a comprehensive clinical examination, which included gathering patient complaints and identifying clinical syndromes associated with encephalopathy following traumatic brain injury. In addition, we evaluated the presence of comorbid conditions, such as cardiovascular diseases, metabolic disorders, and psychiatric conditions, to determine their impact on disease progression.

For cognitive function, Montreal Cognitive Assessment (MoCA) was used. MoCA evaluates multiple cognitive domains, including attention, memory, executive functions, language, and visuospatial skills, with a score of 26 or above considered normal. We stratified patients into three groups based on their MoCA test results: Normal, Mild, and Moderate impairment. In some cases, patients with MoCA scores at the lower limit of the normal range (25 points) were included in the study due to evident impairments in specific cognitive domains, which were not sufficient to significantly affect the total score.

The Barthel Activities of Daily Living (ADL) Index was utilized to evaluate daily functioning. The total score reflects the level of dependence: 0–20 indicates total dependence, 21–60 severe dependence, 61–90 moderate dependence, 91–99 mild dependence, and 100 corresponds to complete independence.

Psychological and psychiatric consultations were carried out to identify behavioral disorders and affective disturbances common in TBI-related encephalopathy

### 2.3. Radiological and Laboratory Examinations

To assess the morphofunctional state of the brain, patients underwent either magnetic resonance imaging (MRI) or computed tomography (CT) (used only in isolated cases when MRI was not feasible due to technical or patient-related limitations, such as the presence of dental braces or intolerance to confined spaces).

A complete blood count was conducted in the morning under fasting conditions prior to the assessment of neurological status. Hematological parameters included RBC, HGB, PLT, WBC, segmented neutrophils, lymphocytes, monocytes, erythrocyte sedimentation rate, and hematocrit levels, to explore potential correlations with clinical symptoms and disease progression.

Molecular–genetic testing was conducted on 26 patients from the general group. The cohort of patients undergoing molecular genetic analysis comprised 92.3% males and 7.7% females. The mean age was (42.2 ± 6.2) years, with an age range of 31 to 55 years. The post-traumatic interval was 1–5 years in 53.8% of cases, 6–10 years in 30.8%, over 10 years in 7.7%, and less than 1 year in 7.7%. Open traumatic brain injury (TBI) was observed in 61.5% of patients, while 38.5% had closed TBI. Regarding injury severity, mild TBI was present in 23.1% of cases, moderate in 53.8%, and severe in 23.1%. The molecular–genetic differentiation of the studied polymorphic gene variants was carried out using allele-specific PCR or PCR-RFLP (restriction fragment length polymorphism) methods, following standard operating protocols developed at the molecular–genetic laboratory of the State Institution “Reference Center for Molecular Diagnostics of the Ministry of Health of Ukraine” (Kyiv). These protocols were based on scientific publications available in open sources [29,30,31] (Table 1).

### 2.4. Statistical Analysis

Power analysis was conducted to ascertain the appropriate sample size necessary to detect statistically significant effects or differences in our study variables, while maintaining a desired level of statistical power. The statistical analysis of the data was conducted using Jamovi software (version 2.2.5; 2022).

For frequency indicators, the absolute number (*n*) and percentage (%) are reported. Pearson’s χ^2^ test was employed to compare frequency characteristics between groups, with a significance level of *p* < 0.05 indicating a statistically significant difference between the studied groups. To compare laboratory findings between groups, we used a nonparametric test, the Kruskal–Wallis test, followed by the Dwass–Steel–Critchlow–Fligner (DSCF) post hoc test.

The development of a prognostic model for the probability of a binary outcome was carried out using logistic regression. Nagelkerke R^2^ was used as a measure of the model performance. ROC analysis was used to assess the diagnostic performance of quantitative variables in predicting a categorical outcome. The optimal cut-off value of the quantitative variable was estimated using Youden’s J statistic.

## 3. Results

### 3.1. Demographic Data

Of the 145 patients included in the study, men constituted the predominant group, comprising 131 individuals (90.3%), whereas women represented a smaller subset, totaling 14 individuals (9.7%). The average age was (42 ± 12.1) years.

Regarding education levels, the majority of individuals had attained a middle-level education (79.3%), while a smaller proportion (20.7%) had achieved a higher level of education.

In order to understand the connection between education level and cognitive impairment, we analyzed the distribution of cognitive impairment across two education groups: “Middle” and “Higher”. Within the “Middle” education group, the majority of individuals exhibited mild cognitive impairment, with 16.5% exhibiting normal cognitive function, 80.0% exhibiting mild impairment, and 3.5% exhibiting moderate impairment. For the “Higher” education group, the distribution was slightly different. Here, 16.7% exhibited normal cognitive function, 76.7% had mild impairment, and 6.6% had moderate impairment.

Among the studied TBIs, 64 patients (44.14%) had open TBI, 76 patients (52.41%) had closed TBI, and in five patients (3.45%) the type of injury was not specified. Regarding the etiological factors, domestic injuries were the most common, observed in 54.48% of patients. Road traffic accidents accounted for 27.59% of cases, military injuries for 11.72%, and occupational injuries were the least frequent at 6.21% (Table 2).

The majority of patients (112, 77.24%) were unemployed. Among the remaining individuals, 16 (11.03%) were engaged in physical labor, and 17 (11.72%) were involved in intellectual work. The disease duration was as follows: up to 1 year, 23 patients; 1–5 years, 44 patients; 6–10 years, 38 patients; and more than 10 years, 40 patients.

### 3.2. Clinical Presentation and Morphometric Changes

Patients with encephalopathy after TBI reported a wide range of complaints. Headache was the most common, affecting 96.55% of patients, followed by dizziness in 58.62%. Tinnitus was noted in 20.69%, and irritability was present in 28.97%. Memory decline was reported by 58.62% of patients, while attention decline was noted in 20.69%. Seizures occurred in 20.00% of patients, and sensory disturbances were observed in 35.86%. Gait disturbances were reported by 21.38%, while sleep disturbances affected 21.38% of individuals. Hearing decline was noted in 26.21% of patients, and vision decline was reported in 15.86%. General weakness was a common complaint, affecting 51.72% of patients. Hypertensive crises were observed in 3.45% of individuals, and pelvic organ dysfunction was reported in 0.69% of patients.

Analyzing the syndromic characteristics of patients with different types of encephalopathies, it was established that patients most frequently presented with the following syndromes: cephalgia (96.55%), emotional liability (73.79%), cerebellar ataxia (34.48%), and pyramidal-reflex insufficiency (46.21%).

According to the Barthel Index, 60.00% of patients were fully independent, 28.28% had mild dependence, and 11.72% had moderate dependence. No cases of severe or complete dependence were recorded.

CT scans of patients with CTE revealed fractures in 55.17% of cases, predominantly at the base of the skull, with most involving a single bone. Neuroimaging identified cysts in 45.05% of patients, mainly in the frontal and temporal regions, with solitary and multiple cysts occurring in nearly equal proportions. Gliosis was observed in 47.59% of cases, while morphometric analysis showed ventricular enlargement in 26.90% and subarachnoid space expansion, indicating external cerebral atrophy, in 38.62% of patients.

### 3.3. Cognitive Functions Assessment

The average score on the Montreal Cognitive Assessment (MOCA) was (23.99 ± 2.40) points, corresponding to mild cognitive impairment. Among patients with encephalopathy after TBI, mild cognitive impairment was identified in 68.28% of individuals, and moderate cognitive impairment in 3.45% of patients, while severe cognitive impairment was not detected. It is noteworthy that the percentage of patients with cephalgia syndrome among those with mild cognitive impairment was 29.29% greater than the number of patients with mild cognitive impairment in whom cephalgia syndrome was not present; however, these differences were statistically improbable (Table 3).

It was established that among patients with CTE diagnosed with ventricular enlargement, the percentage of individuals with moderate cognitive impairment was significantly higher compared to those without ventricular enlargement (10.26% vs. 0.94%, respectively; χ^2^ = 10.60; *p* = 0.005).

To investigate whether blood parameters differ among individuals with varying degrees of cognitive impairment, we applied the Kruskal–Wallis test to a range of hematological indicators across three groups: “mild”, “normal”, and “moderate”. The following results highlight the significant findings and key patterns identified in our analysis. The number of segmented neutrophils significantly differed among the groups (χ^2^ = 16.5508, df = 2, *p* < 0.001). Additionally, lymphocyte counts were significantly different across the groups (χ^2^ = 11.2675, df = 2, *p* = 0.004), indicating a possible connection between lymphocyte levels and cognitive function. Other blood parameters, including red blood cells (RBCs), hemoglobin (HGB), platelets (PLTs), white blood cells (WBCs), monocytes, the erythrocyte sedimentation rate (ESR), and hematocrit (HCT), did not significantly differ, suggesting that these factors may not vary substantially with the level of cognitive impairment (Table 4).

### 3.4. Modeling the Risk of Cognitive Impairments and Functional Impairment in Patients with Encephalopathy After TBI

To achieve the research objective, a logistic regression analysis was conducted, where the dependent binary variable represented the presence (Y = 1) or absence (Y = 0) of cognitive impairments or functional impairment in patients with encephalopathy after TBI. The baseline model for this condition (Model 1) included the polymorphic variants of the studied genes—*ACE*, *AT2R1*, *eNOS*, *PON1*, *IL1β*, *IL10*, and *TNF-α*—as independent variables.

In Model 2, the allelic variants of the genes *ACE*, *AT2R1*, *eNOS*, *PON1*, *IL1β*, *IL10*, and *TNF-α* were included. Model 3 incorporated only prognostically significant genotypes, alongside cofactors such as the patient’s sex (male or female), age category (young: 18–44 years; middle-aged: 45–59 years; elderly: 60–74 years; very elderly: ≥75 years), disease duration (≤1 year, 1–5 years, 6–10 years, >10 years), and the presence or absence of comorbid somatic conditions.

Logistic regression equations were developed to estimate the probability of cognitive impairments and functional impairment in patients with encephalopathies:

Risk of cognitive impairments = 1/(1 + e^x^)

Risk of functional impairment = 1/(1 + e^x^)

where e is the mathematical constant (2.72), and x is a combination of statistically significant predictors.

### 3.5. Analysis of Prognostically Significant Markers for Cognitive Impairment in Patients with Encephalopathy After TBI

Based on logistic regression analysis, the genotypes of *ACE* and the presence of alleles in *ACE* and *AT2R1* genes were identified as prognostically significant factors in the development of cognitive impairments in the patients (Table 5).

For Model 1, the equation for calculating X is as follows:

X = (13.80) + (−5.14) ∗ X1,

where the categorical variable X1 represents the genotype of the *ACE* gene: 1—presence of genotype *I/I*, 2—presence of genotype *I/D*, 3—presence of genotype *D/D*.

Thus, for patients with the *D/D* genotype of the *ACE* gene, the probability of developing cognitive impairments in chronic traumatic encephalopathy is 83.33%. For the *I/I* genotype, the risk is 0.02%, and for the heterozygous *I/D* genotype, the risk is 2.87%.

In the group of patients with encephalopathy after TBI, the formula for calculating the value of X in Model 2 is as follows:

X = (2.53) + (−3.39) ∗ X1 + 2.90⋅X2,

where the categorical variables are defined as follows:

- X1: 1—presence of the I allele of the *ACE* gene, 2—presence of the *D* allele of the *ACE* gene;
- X2: 1—presence of the A allele of the *AT2R1* gene, 2—presence of the *C* allele of the *AT2R1* gene.

Thus, the presence of risk alleles for both genes (*D* allele of the ACE gene and *C* allele of the *AT2R1* gene) results in a 17.48% probability of developing cognitive impairments in studied patients.

Considering that only the *ACE* and *AT2R1* genes were prognostically significant for cognitive impairment development in patients with CTE, Model 3 included only these genes along with the previously mentioned cofactors: patient sex, age category, disease duration, and comorbidities.

It was found that among the proposed markers, the polymorphism of the ACE gene had the highest diagnostic value for patients with these disease (Table 6).

**Figure 1:**
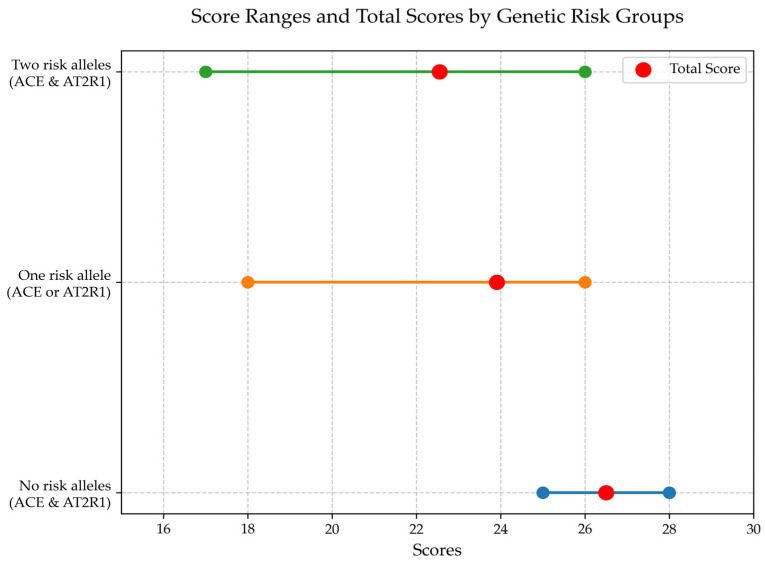
The results of cognitive function analysis in patients with encephalopathy following TBI based on the Montreal Cognitive Assessment depending on the carriage of risk alleles of the ACE and AT2R1 genes.

It was determined that the presence of both risk alleles of the *ACE* and *AT2R1* genes is significantly associated with a reduction in MOCA scores compared to the group of patients who are non-carriers of the risk alleles (Figure 1).

### 3.6. Analysis of Prognostically Significant Markers for Functional Impairment in Patients with Encephalopathy After TBI

In the context of functional impairment development in the studied patients, the polymorphism of the *PON1* gene was identified as prognostically significant. The presence of the T/T genotype of the *PON1* gene was associated with a 41.49% probability of developing functional impairment. Meanwhile, the C/C genotype carried a 24.21% risk, and the heterozygous *C/T* genotype carried a 32.26% risk. The presence of the *T* risk allele of the *PON1* gene alone was associated with a 35.46% probability of functional impairment.

Notably, the inclusion of covariates did not reduce the prognostic significance of the *PON1* genotype concerning the development of functional impairment in patients with this type of encephalopathy (Table 7 and Table 8).

**Figure 2:**
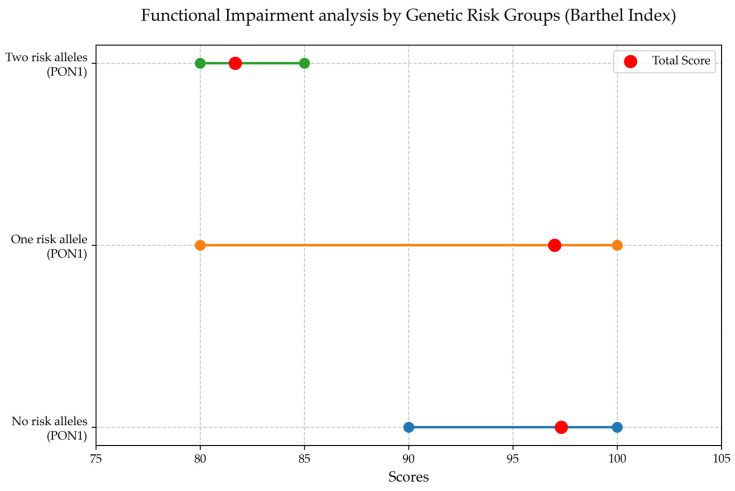
The results of functional impairment analysis in daily life based on the Barthel Index in patients with encephalopathy following TBI depending on the carriage of risk alleles of the PON1 gene.

It should be emphasized that the *T/T* homozygotes of the *PON1* gene exhibit significantly lower Barthel Index scores compared to C/C homozygotes (*p* < 0.05) (Figure 2).

## 4. Discussion

This study examines the complex nature of encephalopathy following traumatic brain injury (TBI), emphasizing the interplay between clinical, neuroimaging, and genetic factors in cognitive dysfunction and functional impairment. The findings align with the existing literature indicating that even mild TBI can result in persistent consequences, including neurodegeneration and chronic encephalopathy [32,33].

### 4.1. Mechanisms Underlying Cognitive and Functional Impairments

The results highlight the role of chronic neuroinflammation and microglial activation in the progression of TBI-related encephalopathy. Structural changes, such as ventricular enlargement and gliosis, observed in this study further support their association with cognitive decline. These mechanisms, reported in prior studies, reflect the multifactorial nature of TBI-related neurodegeneration [34,35].

### 4.2. Genetic Contributions to Disease Progression

This study identified the ACE and AT2R1 gene polymorphisms as important factors associated with cognitive impairment, with the D allele of the ACE gene and the C allele of the AT2R1 gene linked to reduced MOCA scores. Similarly, functional impairment was associated with the T/T genotype of the PON1 gene, which correlated with lower Barthel Index scores [36]. These findings suggest the potential utility of genetic markers in assessing the risk of cognitive and functional decline in TBI patients [37]. However, the variability in the individual contributions of these predictors underscores the need for further research to refine their diagnostic and prognostic value.

The biological mechanisms through which ACE and AT2R1 polymorphisms influence cognitive outcomes following TBI may involve dysregulation of the renin–angiotensin system (RAS). Altered expression or activity of these genes can contribute to increased neuroinflammation, oxidative stress, and blood–brain barrier disruption, thereby promoting neuronal damage and impaired cognitive recovery. Given these mechanistic links, ACE and AT2R1 genotypes may not only serve as risk factors but also represent promising prognostic biomarkers for post-traumatic encephalopathy.

### 4.3. Clinical and Functional Implications

Cognitive impairments and functional limitations were prevalent among the study cohort, significantly impacting quality of life. Mild cognitive impairment was observed in 68.28% of patients, and moderate functional dependence was noted in 11.72%. These findings are consistent with previous studies and highlight the substantial burden of TBI on patients and caregivers [38,39]. Even individuals classified as functionally independent reported difficulties in social reintegration and employment, reflecting the broader impact of TBI beyond physical recovery [40].

## 5. Conclusions

This study underscores the importance of integrating clinical, neuroimaging, and genetic data in assessing cognitive and functional impairments in TBI patients. The identified associations between genetic polymorphisms and clinical outcomes highlight their potential role in improving risk stratification and guiding personalized approaches to management. Expanding the scope of future research to include larger cohorts and longitudinal designs could further elucidate the mechanisms of TBI-related neurodegeneration and support the development of targeted diagnostic and therapeutic strategies.

## 6. Limitations and Future Directions

This study has several limitations that should be noted. First, the overall sample size was relatively small, particularly for the subgroup involved in the genetic analyses. However, a power analysis was conducted prior to the study, and results indicated that the sample size was sufficient to detect statistically significant effects within the scope of our primary objectives. Nevertheless, the limited number of participants may still restrict the broader generalizability of our findings. Second, the study was carried out at a single center, which might introduce biases related to local patient demographics and treatment protocols. Finally, the lack of longitudinal follow-up prevents us from assessing the long-term trajectory of cognitive and functional outcomes following traumatic brain injury.

## Figures and Tables

**Table 1:** Molecular–genetic differentiation of the studied gene variants.

No.	Gene	International Name of Genetic Polymorphism, rs	Reference to the Source
1.	*IL-1ß*	*C3953T*, *g.8967C>T*, *rs1143634*	[16]
2.	*IL-10*	*C-592A*, *g.4433A>C*, *rs1800872*	[17]
3.	*TNFα*	*G308A*, *c.-488G>A*, *rs180062*	[18]
4.	*ACE*	*I/D*, *c.2306-117_2306-116insAF118569.1:g.14094_14382*, *rs4340*	[18]
5.	*eNOS*	*4a/4b*, *g.150997188 AGGGGTGAGGAAGT CTAGACCTGCTGC [1]*, *rs61722009*	[19]
6.	*PON1*	*C108T*, *g.5124C>T*, *rs705379*	[16]
7.	*AT2R1*	*A1166C*, *g.148742201A>C*, *rs5186*	[18]

**Table 2:** Distribution of types and severity of traumatic brain injury (TBI).

Severity of TBI	*n* (%)	Type	*n* (%)
Mild	35 (24.14)	Concussion	20 (57.14)
		Contusion	15 (42.86)
Moderate	90 (62.07)	Contusion + SAH, epidural or subdural hemorrhage, fractures	54 (60.00)
		Contusion + intracerebral hemorrhage, fracture	7 (7.78)
		Contusion + fracture	29 (32.22)
Severe	20 (13.79)	Severe contusion, diffuse axonal injury, brain compression	20 (100.00)

**Table 3:** Evaluation of cognitive functions in patients with encephalopathy after TBI based on the results of Montreal Cognitive Assessment (MoCA) analysis depending on dominant clinical syndromes.

Clinical Syndromes		Normal Cognitive Functioning		Cognitive Impairment						χ^2^; *p*
				Mild		Moderate		Severe		
		*n*	%	*n*	%	*n*	%	*n*	%	
Cephalgia	−	3	60.00	2	40.00	0	0	0	0	χ^2^ = 2.63; *p* = 0.269
	+	38	27.14	97	69.29	5	3.57	0	0	
Emotional-liability syndrome	−	12	31.58	23	60.53	3	7.89	0	0	χ^2^ = 3.60; *p* = 0.165
	+	29	27.10	76	71.03	2	1.87	0	0	
Cerebellar ataxia	−	29	30.53	62	65.26	4	4.21	0	0	χ^2^ = 1.32; *p* = 0.516
	+	12	24.00	37	74.00	1	2.00	0	0	
Pyramidal insufficiency	−	20	25.64	54	69.23	4	5.13	0	0	χ^2^ = 1.82; *p* = 0.403
	+	21	31.34	45	67.16	1	1.49	0	0	

Note 1: − absence of the syndrome; + presence of the syndrome.

**Table 4:** Evaluation of cognitive functions in patients with encephalopathy after TBI based on the results of Montreal Cognitive Assessment (MOCA) analysis depending on hematological parameters.

Indicators	Cognitive Impairment			*p*
	Norm	Mild	Moderate	
RBC (×10^12^/L)	5.10	4.95	4.30	0.051
	(4.68–5.39)	(4.70–5.28)	(4.20–4.67)	
HGB (g/L)	152	148	152	0.622
	(150–154)	(141–158)	(150–154)	
PLT (×10⁹/L)	218	214	231	0.944
	(196–236)	(189–254)	(190–272)	
WBC (×10⁹/L)	5.92	6.47	5.77	0.598
	(5.25–7.01)	(5.10–7.60)	(5.54–7.07)	
Segmented Neutrophils (%)	51	60	47	<0.001 *
	(46.8–57.3)	(54.00–65.0)	(41.0–52.0)	
Lymphocytes (%)	36.0	30.00	36.0	0.004 *
	(30.0–40.0)	(22.0–35.9)	(32.9–38.0)	
Monocytes (%)	7.00	7.00	7.10	0.958
	(4.00–9.00)	(5.00–8.00)	(2.00–7.50)	
Erythrocyte Sedimentation Rate (mm/hr)	5.00	5.00	3.00	0.475
	(3.00–10.0)	(3.00–11.0)	(2.00–7.00)	
Haematocrit (%)	46.5	45.5	46.8	0.706
	(43.2–48.1)	(42.7–48.0)	(44.8–48.2)	

*—statistically significant result. Median (IQR).

**Table 5:** Results of logistic regression analysis on the development of cognitive impairments in patients with encephalopathy after TBI.

Factor	Model 1				Model 2			
	Genotypes				Alleles			
	β	SE	t	*p*	β	SE	t	*p*
Constant	13.80	11.67	1.18	0.010 *	2.53	2.40	1.06	0.007 *
*ACE*	−5.14	2.21	−2.32	0.032 *	−3.39	1.32	−2.57	0.013 *
*AT2R1*	1.78	1.37	1.30	0.211	2.90	1.38	2.11	0.041 *
*eNOS*	−4.53	3.64	−1.24	0.230	−2.49	1.54	−1.61	0.114
*IL1β*	1.21	1.66	0.73	0.473	0.82	1.48	0.55	0.582
*TNFα*	1.63	2.42	0.67	0.509	0.29	1.55	0.19	0.853
*IL10*	−2.85	2.44	−1.17	0.258	−0.26	1.27	−0.20	0.839
*PON1*	−1.27	1.17	−1.09	0.291	−0.37	0.87	−0.43	0.67

Note 1. β—logistic regression coefficient; SE—standard error; t—Wald test statistic; *p*—significance level. Note 2. *—statistically significant result.

**Table 6:** Results of logistic regression analysis on the development of cognitive impairments in patients with encephalopathy after TBI considering gender, age category, presence of somatic comorbidities, and disease duration.

Factor	Model 3			
	β	SE	t	*p*
Constant	−0.42	0.64	−0.66	0.046 *
*ACE*	0.33	0.12	2.68	0.015 *
*AT2R1*	−0.16	0.13	−1.19	0.250
Sex	0.17	0.38	0.46	0.654
Age category	0.12	0.21	0.55	0.587
Somatic comorbidity	0.25	0.28	0.89	0.385
Disease duration	0.13	0.11	1.12	0.276

Note 1. β—logistic regression coefficient; SE—standard error; t—Wald test statistic; *p*—significance level. Note 2. *—statistically significant result.

**Table 7:** Results of logistic regression analysis on the development of functional disability in daily life in patients with encephalopathy after TBI.

Factor	Model 3				Model 2			
	β	SE	t	*p*	β	SE	t	*p*
Constant	1.54	0.99	1.55	0.037 *	1.56	0.37	4.24	0.001 *
*ACE*	0.07	0.14	0.53	0.603	0.19	0.16	1.23	0.224
*AT2R1*	−0.10	0.17	−0.58	0.570	0.10	0.17	0.59	0.561
*eNOS*	0.38	0.29	1.33	0.200	0.10	0.19	0.53	0.598
*IL1β*	0.05	0.23	0.22	0.828	−0.00	0.23	−0.02	0.983
*TNFα*	0.02	0.27	0.08	0.935	−0.01	0.22	−0.03	0.978
*IL10*	−0.03	0.28	−0.10	0.920	−0.02	0.19	−0.12	0.903
*PON1*	−0.40	0.14	−2.84	0.011 *	−0.48	0.15	−3.14	0.003 *

Note 1. β—logistic regression coefficient; SE—standard error; t—Wald test statistic; *p*—significance level. Note 2. *—statistically significant result.

**Table 8:** Results of logistic regression analysis on the development of functional disability in daily life in patients with encephalopathy after TBI, disease duration.

Factor	Model 3			
	β	SE	t	*p*
Constant	1.91	0.65	2.96	0.008 *
*PON1*	−0.42	0.13	−3.39	0.003 *
Sex	0.37	0.36	1.02	0.321
Age category	−0.05	0.20	−0.24	0.815
Somatic comorbidity	−0.07	0.11	−0.65	0.521
Disease duration	0.34	0.28	1.21	0.241

Note 1. β—logistic regression coefficient; SE—standard error; t—Wald test statistic; *p*—significance level. Note 2. *—statistically significant result.

## Data Availability

The original contributions presented in the study are included in the article, further inquiries can be directed to the corresponding authors.
